# TRPM8 Activation via 3-Iodothyronamine Blunts VEGF-Induced Transactivation of TRPV1 in Human Uveal Melanoma Cells

**DOI:** 10.3389/fphar.2018.01234

**Published:** 2018-11-13

**Authors:** Lia Walcher, Clara Budde, Arina Böhm, Peter S. Reinach, Priyavathi Dhandapani, Nina Ljubojevic, Markus W. Schweiger, Henriette von der Waydbrink, Ilka Reimers, Josef Köhrle, Stefan Mergler

**Affiliations:** ^1^Klinik für Augenheilkunde, Charité – Universitätsmedizin Berlin, Corporate Member of Freie Universität Berlin, Berlin Institute of Health, Humboldt-Universität zu Berlin, Berlin, Germany; ^2^School of Ophthalmology and Optometry, Wenzhou Medical University, Wenzhou, China; ^3^MDC Buch, Berlin, Germany; ^4^Institut für Experimentelle Endokrinologie, Charité – Universitätsmedizin Berlin, Corporate Member of Freie Universität Berlin, Berlin Institute of Health, Humboldt-Universität zu Berlin, Berlin, Germany

**Keywords:** uveal melanoma, 3-iodothyronamine, vascular endothelial growth factor, Intracellular Ca^2+^, transient receptor potential vanilloid 1 channel, transient receptor potential melastatin 8

## Abstract

In human uveal melanoma (UM), tumor enlargement is associated with increases in aqueous humor vascular endothelial growth factor-A (VEGF-A) content that induce neovascularization. 3-Iodothyronamine (3-T_1_AM), an endogenous thyroid hormone metabolite, activates TRP melastatin 8 (TRPM8), which blunts TRP vanilloid 1 (TRPV1) activation by capsaicin (CAP) in human corneal, conjunctival epithelial cells, and stromal cells. We compare here the effects of TRPM8 activation on VEGF-induced transactivation of TRPV1 in an UM cell line (92.1) with those in normal primary porcine melanocytes (PM) since TRPM8 is upregulated in melanoma. Fluorescence Ca^2+^-imaging and planar patch-clamping characterized functional channel activities. CAP (20 μM) induced Ca^2+^ transients and increased whole-cell currents in both the UM cell line and PM whereas TRPM8 agonists, 100 μM menthol and 20 μM icilin, blunted such responses in the UM cells. VEGF (10 ng/ml) elicited Ca^2+^ transients and augmented whole-cell currents, which were blocked by capsazepine (CPZ; 20 μM) but not by a highly selective TRPM8 blocker, AMTB (20 μM). The VEGF-induced current increases were not augmented by CAP. Both 3-T_1_AM (1 μM) and menthol (100 μM) increased the whole-cell currents, whereas 20 μM AMTB blocked them. 3-T_1_AM exposure suppressed both VEGF-induced Ca^2+^ transients and increases in underlying whole-cell currents. Taken together, functional TRPM8 upregulation in UM 92.1 cells suggests that TRPM8 is a potential drug target for suppressing VEGF induced increases in neovascularization and UM tumor growth since TRPM8 activation blocked VEGF transactivation of TRPV1.

## Introduction

Among all cancers of the eye, uveal melanoma (UM) is the most frequent form in adults. Notably, UM is mostly found in the choroid (65% of all cases) and in ciliary body (15%), but it rarely occurs in the retina (1.4%; Singh et al., 2011). About 50% of the patients with primary UM will finally develop distant metastases predominantly in the liver (90%) (Spagnolo et al., 2012). To date, the etiology of UM is not fully understood and neither metastatic properties nor patient survival has significantly improved over the last decades (Tran et al., 2013). Accordingly, there is a pressing need for developing alternative approaches to treat this disease especially since there are no FDA approved drugs available for suppressing metastatic melanoma.

A preclinical approach targeting angiogenesis in combination with irradiation has been reported using bevacizumab, a monoclonal antibody binding and inhibiting vascular endothelial growth factor (VEGF; Sudaka et al., 2013). Nevertheless, the advantage of this combination therapy is unclear because this VEGF trap did not have a dramatic impact on any of the functional activities in UM cell lines (Logan et al., 2013). As a matter of fact, such treatment is reported to even promote expansion of melanoma cells *in vitro* (Dithmer et al., 2017). Furthermore, neoadjuvant intravitreous injection of this VEGF trap failed to shrink large size melanoma and is even counter indicated in these cases because it may instead even promote melanoma growth (Francis et al., 2017).

Increases in VEGF receptor activity induce rises in intracellular calcium levels [Ca^2+^]_i_ in endothelial cells exposed to serum-free conditioned medium of human malignant gliomas (Criscuolo et al., 1989). The bioactive factor is an angiogenic factor named vascular permeability factor (VPF)—more recently characterized as VEGF, which promotes various diseases including eye tumor diseases (e.g., retinoblastoma) (Jia et al., 2007). It stimulates angiogenesis through activating non-voltage-gated Ca^2+^ channels such as transient-receptor-potential-channels (TRPs) namely the canonical receptor type 4 or 6 (TRPC4 or TRPC6) in human microvascular endothelial cells (Qin et al., 2016). Dysfunctional TRPs are implicated in cancer formation (reviewed in Bödding, 2007; Prevarskaya et al., 2007). Tumor and normal cells both express TRPs, but certain TRPs are either upregulated or downregulated in a cancerous condition. For example, TRP vanilloid receptor type 1 (TRPV1; capsaicin receptor) is overexpressed in some carcinomas (Miao et al., 2008; Marincsák et al., 2009) and neuroendocrine tumors (Mergler et al., 2012b). In addition, the highly Ca^2+^ selective TRPV6 and TRP melastatin receptor type 8 (TRPM8; menthol receptor) are overexpressed in prostate tumor cells (Fixemer et al., 2003; Bidaux et al., 2005; Bai et al., 2010; Gkika et al., 2010). The functional relevance of TRPM8 upregulation in prostatic cancer cells as a target for suppressing their proliferation was documented by showing that inhibition of TRPM8 upregulation with highly specific blockers, AMTB, JNJ41876666, and RNAi suppressed increased proliferation rates in all tumor cells but not in non-tumor prostate cells (Valero et al., 2012). We found that TRPM8 is also overexpressed in highly malignant retinoblastoma and uveal melanoma along with TRPV1 compared to their levels in healthy human uvea or retina (Mergler et al., 2012a, 2014). Even in benign pterygial eye tumor cells, functional TRPV1 expression is upregulated (Garreis et al., 2016). Such increases are associated with larger mitogenic responses to VEGF that are induced by its cognate receptor, VEGFR, transactivating TRPV1 (Garreis et al., 2016).

3-iodothyronamine (3-T_1_AM) is a decarboxylated thyroid hormone (T_3_ and T_4_) metabolite, which activates G protein-coupled receptors (GPCRs) especially the trace amine associated receptor 1 (TAAR1). It also induces a dose-dependent reversible 10°C decrease in mice body temperature (Scanlan et al., 2004; Braulke et al., 2008; Panas et al., 2010) and hypothermia in rodents (Cichero et al., 2014; Hoefig et al., 2016). Likewise, 3-T_1_AM is a multi-target ligand modulating β-adrenergic receptor 2 signaling in ocular epithelial cells (Dinter et al., 2015a). In corneal epithelial and endothelial cells as well as thyroid cells, 3-T_1_AM acts as a selective TRPM8 agonist (Khajavi et al., 2015, 2017; Lucius et al., 2016; Schanze et al., 2017). Since blocking increases in VEGF levels suppress both angiogenesis and expansion of tumorous pathology, it is relevant to identify novel targets to inhibit endothelial cell proliferation. We hypothesized that TRPM8 is one such target because icilin-induced TRPM8 activation suppressed TRPV1 activity in cornea and conjunctiva epithelial cells (Khajavi et al., 2015; Lucius et al., 2016). The notion that TRPM8 activation also inhibits VEGF-induced TRPV1 activation required for increasing angiogenesis was tenable because VEGF-induced activation of its cognate receptor transactivates TRPV1 (Khajavi et al., 2015; Lucius et al., 2016).

We show here that crosstalk between members of this receptor triad affects Ca^2+^ signaling responses induced by VEGFR transactivation of TRPV1 in UM 92.1 melanoma cells. Therefore, selective targeting of TRPM8 control of TRPV1 responsiveness to transactivation by VEGF may ultimately provide an alternative approach to reduce tumor growth in a clinical setting.

## Materials and methods

### Materials

BCTC, AMTB, and fura-2AM were purchased from TOCRIS Bioscience (Bristol, United Kingdom). CPZ and icilin were procured from Cayman Chemical Company (Ann Arbor, Michigan, U.S.A.). Medium and supplements for cell culture were ordered from Life Technologies Invitrogen (Karlsruhe, Germany) or Biochrom AG (Berlin, Germany). Melanocyte Growth Medium M2 was obtained from Promocell (Heidelberg, Germany). Dispase II was ordered from Boehringer (Ingelheim, Germany) and accutase was provided by PAA Laboratories (Pasching, Austria). Unless otherwise stated, all other reagents were procured from Sigma (Deisenhofen, Germany).

### Cell culture

Uveal melanoma cell line 92.1 (UM 92.1) was kindly provided by Martine Jager and colleagues (Leiden University; Netherlands) (De Waard-Siebinga et al., 1995). In brief, UM cells were grown in RPMI-1640 supplemented with 10% fetal bovine serum (FBS), 4 mM L-glutamine, penicillin/streptomycin at 37°C under 10% CO_2_ atmosphere and 80% humidity (Mergler et al., 2014).

### Melanocyte primary cell cultivation

PM were isolated from porcine eyes provided by a slaughterhouse. The preparation and primary cell cultivation were performed as described (Valtink and Engelmann, 2007). In brief, eyeballs were cut into two pieces. The choroid with the connected retinal pigment epithelium (RPE) layer was separated from the sclera and incubated in collagenase IV for several hours at 37°C in order to release RPE cells from melanocytes. After a second treatment with dispase II, the choroids were put into a shaking device in order to better isolate the cells from the tissue. Finally, the cell suspension was passed through a cell strainer. After centrifugation, cells were washed in RPMI medium and seeded in tissue culture flasks. After 24 h, the medium was changed and cells were cultivated under the same conditions as those described for the UM 92.1 cells (Mergler et al., 2014). To avoid contamination with RPE cells or fibroblasts, the culture medium was supplemented with geneticin for about 5–7 days prior to subcultivation. Melanocyte cell cultivation was limited to no longer than 2 weeks to avoid cell dedifferentiation.

### Intracellular calcium fluorescence imaging

Semi confluent cells (≈80%) were loaded with fura-2/AM (2 μM) at 37°C. After about 40 min, the cells were washed with a Ringer-like (control) solution containing (mM): 150 NaCl, 6 CsCl, 1 MgCl_2_, 10 glucose, 10 HEPES, and 1.5 CaCl_2_ at pH 7.4 and 317 mOsM (Mergler et al., 2014). KCl was replaced with CsCl to characterize TRP channel activity (Voets et al., 2004). Following dye loading, the cells were exposed to this solution on the stage of an inverted microscope (Olympus BW50WI, Olympus Europa Holding GmbH, Hamburg, Germany), connected with a digital imaging system (TILL Photonics, Munich, Germany). Fura-2/AM fluorescence was consecutively excited at 340 and 380 nm for different times (Grynkiewicz et al., 1985). The 510 nm emission ratio (f_340nm_/f_380nm_) is an index of relative intracellular Ca^2+^ ([Ca^2+^]_i_) levels (Grynkiewicz et al., 1985). The 340 and 380 nm response signals were continuously detectable and did not distort the ratio. The changes in ratios were overall small because of the presetting of the single fluorescence signals at 340 and 380 nm, respectively. A control where TRPM8 was heterologously expressed and activated by their agonists is provided (Lucius et al., 2016). Before starting a measuring session, baseline stability was established for 8–20 min. All experiments were performed at a constant room temperature (≈23°C). In addition, the fura-2-induced fluorescence signals were alternatively evaluated in a bath chamber using a Life Science fluorescence cell imaging software in conjunction with a high-resolution digital camera (Olympus XM-10) (**Figures 9–11**). Cutoff filters isolated alternative fluorescence excitation at 340 and 380 nm every 5 s wavelengths provided by a LED light source (LED-Hub by Omikron, Rodgau-Dudenhoven, Germany). Fura-2 fluorescence was alternately excited at 340 and 380 nm and emission was detected at 510 nm (250 ms−3.8 s exposure time). For image acquisition and data evaluation, the Life Science imaging software cellSens was used (Olympus, Hamburg, Germany). Results are shown as mean traces of the f_340nm_/f_380nm_ ratio ± SEM (error bars in both directions) with n*-*values indicating the number of experiments per data point. The Ca^2+^ data presented from many cells in several experiments were normalized (control set to 1.2 and 0.2, respectively) and averaged (with error bars). The time delay of 1–2 min in inducing a Ca^2+^ transient stems from exposing the cells to a stationary bath rather than a flow through superfusion. When drugs were solubilized in dimethyl sulfoxide (DMSO) solution, their working concentration did not exceed 0.1%, which did not alter the Ca^2+^ base line.

### Planar patch-clamp recordings

Whole-cell currents were measured with a planar patch-clamp setup (Port-a-Patch^©^; Nanion, Munich, Germany) in connection with an EPC 10 patch-clamp amplifier (HEKA, Lamprecht, Germany) and the PatchMaster software (Version 2.6; HEKA, Lamprecht, Germany) (Mergler et al., 2012a, 2014; Garreis et al., 2016). A standard intracellular solution containing (mM): 50 CsCl, 10 NaCl, 60 CsF, 20 EGTA, and 10 HEPES-acid at pH ≈ 7.2 and ≈ 288 mOsM was applied to the microchip (both provided by Port-a-Patch^©^, Nanion, Munich, Germany). The external solution contained (mM): 140 NaCl, 4 KCl, 1 MgCl_2_, 2 CaCl_2_, 5 D-glucose monohydrate and 10 HEPES, pH ≈ 7.4, and osmolarity ≈ 298 mOsM. At first, 5–10 μl of a single cell suspension were placed onto a microchip containing the aforementioned external solution. A software-controlled pump (Nanion) provided a connection between a single cell and the electrical system (sealing). The mean membrane capacitance was 10 pF ± 1 pF (*n* = 88) and mean access resistance was 25 ± 3 MΩ (*n* = 88). Series resistances as well as fast and slow capacitative transients were compensated by the PatchMaster software. The liquid junction potential was calculated (≈3.8 mV; Barry, 1994) and offset by the software. Notably, current recordings were all leak-subtracted and cells with leak currents above 100 pA were excluded from analysis. All experiments were performed at 21–23°C room temperature. The holding potential (HP) was set to 0 mV in order to eliminate any possible contribution of voltage-dependent Ca^2+^ channel activity. Cells were kept in the whole-cell configuration for ~10 min for control recordings and the compensation proceedings before starting the experiments (Pusch and Neher, 1988). Whole-cell currents were recorded over a voltage range of −60 to +130 mV for 500 ms each and measured every 5 s. The current densities (pA/pF) were calculated by dividing the current (pA) by the cell membrane capacitance (pF). For purposes of comparison, the currents were normalized to control currents (set to 100%).

### Statistical analysis

The paired two-tailed Student's *t*-test was applied in conjunction with several normality tests (KS normality test, D'Agostino & Pearson omnibus normality test, and Shapiro-Wilk normality test). If these tests failed, non-parametric Wilcoxon matched pairs were used. The Student's *t*-test was also used for unpaired data, if the data also passed the aforementioned normality tests. If these tests failed, the non-parametric Mann-Whitney-*U* test was performed. Welch's correction was applied if data variance of the two groups were too different. Probabilities of *p* < 0.05 [indicated by asterisks (^*^) and hash tags (#)] were considered to be significant. The number of repeats is shown in each case in brackets, near the traces or bars. All values are means ± SEM (error bars in both directions). All plots were generated with SigmaPlot software version 12.5 for Windows (Systat Software, San Jose, California, United States) and with GraphPad Prism version 5.00 for Windows (GraphPad Software, San Diego California USA), respectively.

## Results

### Functional TRPV1 channel expression in UM 92.1 and porcine melanocytes

**Figure 1:**
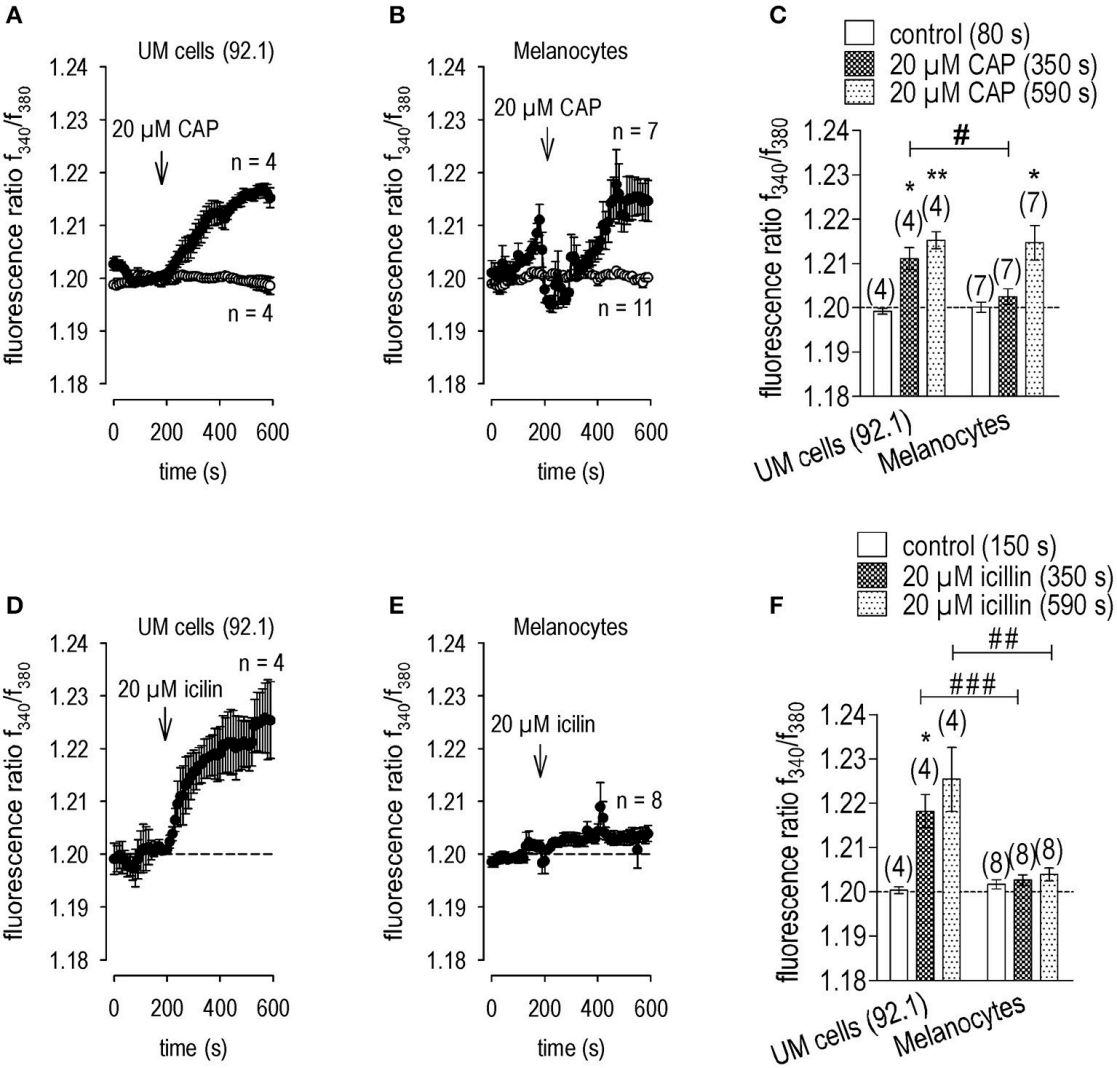
Larger functional TRPM8 expression in UM 92.1 than PM cells. Drug additions were made at the indicated time points (arrows). Data are mean ± SEM of 4–8 experiments. **(A)** CAP (20 μM) induced an irreversible Ca^2+^ influx (*n* = 4) whereas non-treated control cells showed a constant Ca^2+^ baseline (*n* = 4). **(B)** The same effect could be observed in normal porcine melanocytes but with a time lag (*n* = 7; controls *n* = 11). **(C)** Summary of the experiments with CAP (*n* = 4–7). The asterisks (*) show significant differences between control and CAP (*n* = 4; 350s; *p < 0.05; *n* = 7; 590 s; **p < 0.01; paired tested). The hashtag shows a significant difference of the CAP effect at 350 s between UM 92.1 cells and melanocytes (*n* = 4-7; 590 s; ^#^*p* < 0.05; unpaired tested). **(D–F)** Same experiments as shown in **(A–C)**, but with icilin (20 μM) (*n* = 4–8). The icilin effect was markedly reduced in porcine melanocytes. The asterisks (*) show significant differences between control and icilin (*n* = 4; 350 s; *p < 0.05; paired tested). The hashtags (#) shows a significant difference of the icilin effect at 350 s and 590 s between UM 92.1 cells and melanocytes (*n* = 4–8; 350 s; ^*###*^*p* < 0.005; 590 s; ^*##*^*p* < 0.01; unpaired tested).

TRPV1 activity in all cases was documented based on the magnitudes of the Ca^2+^ transients induced by the highly selective TRPV1 agonist, capsaicin (CAP; 20 μM; Caterina et al., 1997; Pingle et al., 2007; Vriens et al., 2009) in PM. Figure 1B shows that CAP increased f_340nm_/f_380nm_ from a stable baseline value of 1.200 ± 0.001 to 1.215 ± 0.004 after 590 s (*n* = 7, *p* < 0.05). Interestingly, CAP evoked a biphasic or delayed effect on intracellular Ca^2+^ increase in PM, which was absent in UM 92.1 tumorous cells. In UM 92.1 cells, CAP instead increased the f_340nm_/f_380nm_ ratio more promptly but to the same level; namely, from 1.199 ± 0.001 (80 s) to 1.215 ± 0.002 after 590 s (*n* = 4, *p* < 0.01; Figures 1A,C). Overall, there is a difference in the Ca^2+^ response pattern. While in PM, there was a large data scatter and a delayed [Ca^2+^]_i_ transient (Figure 1B), this response was both more reproducible and prompt in UM 92.1 cells (Figure 1A).

### Functional TRPM8 channel expression in UM 92.1 cells

Even though there was TRPM8 gene and functional expression in different UM cell lines including UM 92.1 cells, it was absent in human uveas (Mergler et al., 2014). To confirm that lack of TRPM8 expression is indicative of normal tissue, we probed for its presence in healthy PM. Icilin (20 μM), a mixed TRPM8/ TRPA1-agonist (Andersson et al., 2004; Rawls et al., 2007) induced a Ca^2+^ transient in UM 92.1 cells (*n* = 4; *p* < 0.05; Figures 1D,F) whereas such an effect did not occur in PM (*n* = 8; *p* > 0.05; Figures 1E,F). Therefore, detectable functional TRPM8 and/or TRPA1 expression is a marker of UM cell line malignancy.

### VEGF-transactivates TRPV1

**Figure 2:**
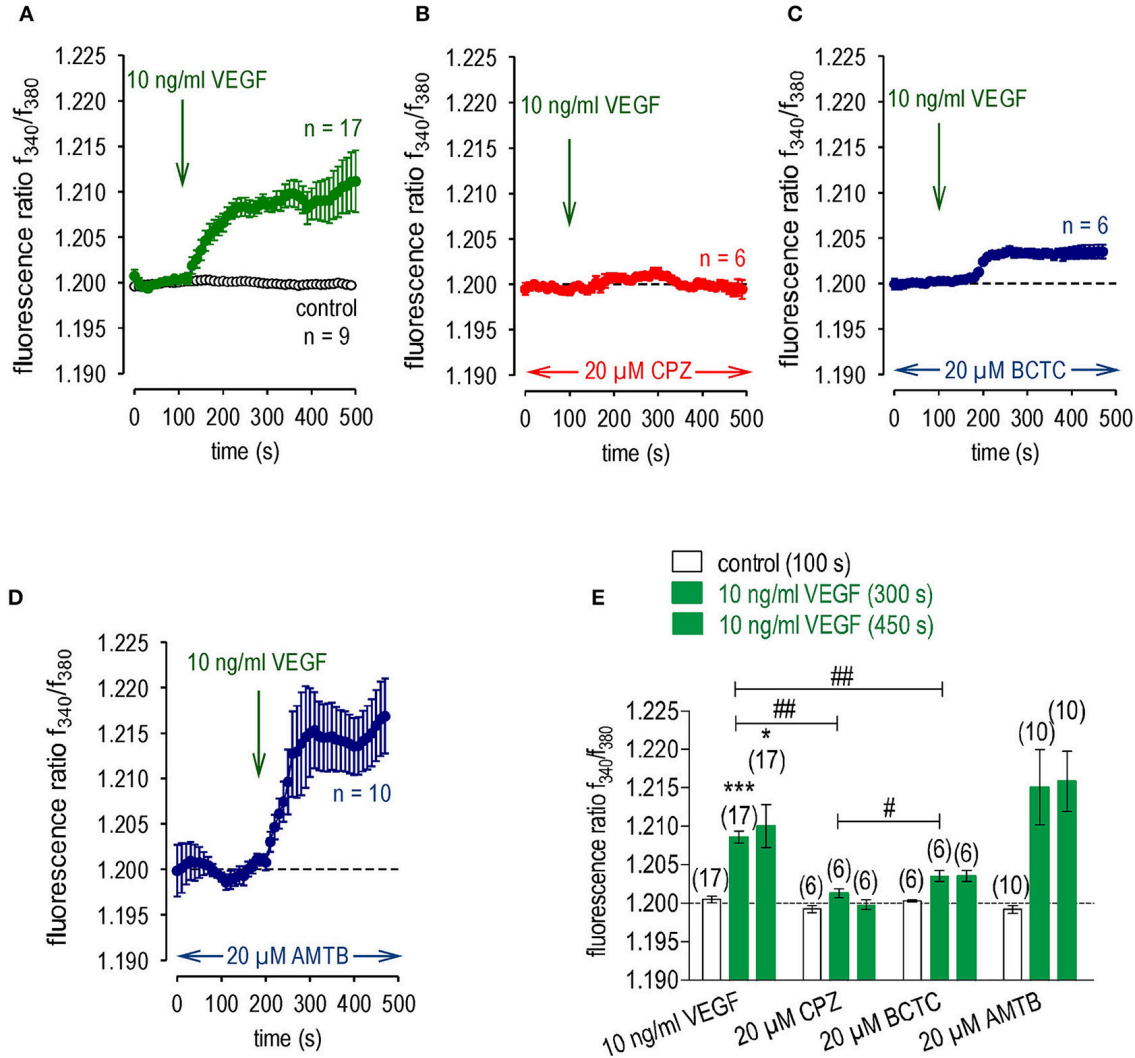
VEGF transactivates TRPV1 channels in UM 92.1 cells. VEGF (10 ng/ml) was added at the indicated time points (arrows). Data are mean ± SEM of 6–17 experiments. **(A)** Mean trace showing VEGF-induced Ca^2+^ increase (*n* = 17). **(B)** Same experiment as shown in **(A)**, but in the presence of CPZ (20 μM). CPZ clearly suppressed the VEGF-induced Ca^2+^ increase (*n* = 6). **(C)** Same experiment as shown in **(A)**, but in the presence of BCTC (20 μM). BCTC partially suppressed the VEGF-induced Ca^2+^ increase (*n* = 6). **(D)** Same experiment as shown in **(A)**, but in the presence of AMTB (20 μM). AMTB had no effect on the VEGF-induced Ca^2+^ increase (*n* = 10). **(E)** Summary of the experiments with VEGF and the TRP channel blockers. The asterisks (*) show significant Ca^2+^ increases with VEGF (*n* = 17; 300 s; ***p < 0.005; 450 s; *p < 0.05; paired tested). The hashtags (##) indicate statistically significant differences of fluorescence ratios between VEGF with and without the TRP channel blockers CPZ and BCTC, resp. (*n* = 6–17; 300 s; ^*##*^*p* < 0.01; unpaired tested). One hashtag (#) indicates a statistically significant difference between CPZ and BCTC effect on VEGF-induced Ca^2+^ increase at 300 s (*n* = 6; ^#^*p* < 0.05).

VEGF increased the f_340nm_/f_380nm_ ratio from 1.2000 ± 0.0004 to 1.209 ± 0.001 (*t* = 300 s; *n* = 17; *p* < 0.01, Figures 2A,E). As VEGF induced Ca^2+^ transients through transactivating TRPV1 channels in corneal fibroblasts and conjunctival epithelial cells, we determined if such an interaction occurs in UM 92.1 cell lines. This was done by evaluating the individual effects of each of the following inhibitors at 20 μM: (a) CPZ for TRPV1 (Vriens et al., 2009); (b) BCTC for TRPM8/TRPV1 (Behrendt et al., 2004; Weil et al., 2005; Vriens et al., 2009; Benko et al., 2012; Liu et al., 2016); and (c) AMTB for TRPM8 (Lashinger et al., 2008). With CPZ, the baseline ratio remained invariant at f_340nm_/f_380nm_ ratio = 1.2013 ± 0.0006 (*n* = 6; Figure 2B) whereas with AMTB this ratio rose to 1.215 ± 0.005; *p* > 0.05; *n* = 10; Figure 2D. This difference indicates that the VEGF-induced Ca^2+^ transients mediated by VEGFR solely transactivate TRPV1. With BCTC, the VEGF induced Ca^2+^ transients were only partially inhibited (Figure 2C). In this case, VEGF induced a transient reaching 1.2030 ± 0.0005 (*p* < 0.01; *n* = 6 Figure 2C), which was larger than the ratio induced by VEGF in the presence of CPZ (*p* < 0.05; *n* = 6; Figures 2B,D). This difference is consistent with BCTC being a mixed TRPM8/TRPV1 antagonist.

**Figure 3:**
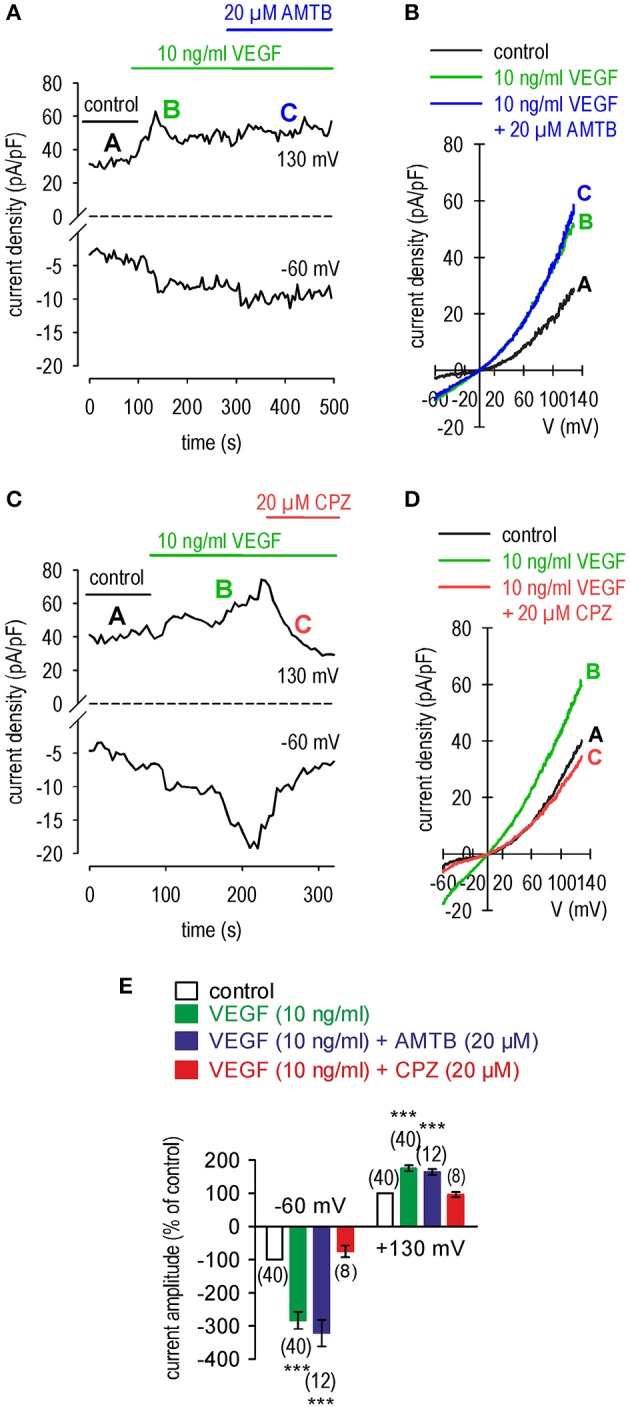
Comparison of the effects of CPZ and AMTB on VEGF-induced rises in whole-cell currents in UM 92.1 cells. **(A)** Time course recording showing the current increases induced by VEGF (10 ng/ml) and after application of 20 μM AMTB. **(B)** Original traces of VEGF-induced current responses to voltage ramps. Current densities are shown before application (labeled as **A**), during application of VEGF (labeled as **B**), and after addition of AMTB (labeled as **C**). **(C–D)** Same recordings as shown in **(A–B)** but with 20 μM CPZ instead of AMTB. CPZ clearly reduced the VEGF-induced whole-cell increases. **(E)** Summary of patch-clamp experiments with VEGF, AMTB and CPZ. The asterisks (***) indicate statistically significant differences of in- and outward currents with VEGF without and with AMTB (*n* = 8–40; ***p < 0.005; paired tested). VEGF had no effect in the presence of CPZ.

While AMTB did not influence the VEGF-induced increases in whole-cell currents (20 μM; *n* = 12; Figures 3A,B,E), this increase was suppressed by CPZ (20 μM; *n* = 8; *p* < 0.005; Figures 3C–E). In summary, the VEGF-induced increases in Ca^2+^ influx and whole-cell currents are mediated through transactivation of TRPV1 by VEGFR.

### Equivalent activation of TRPV1 by VEGF and CAP in UM 92.1 cells

**Figure 4:**
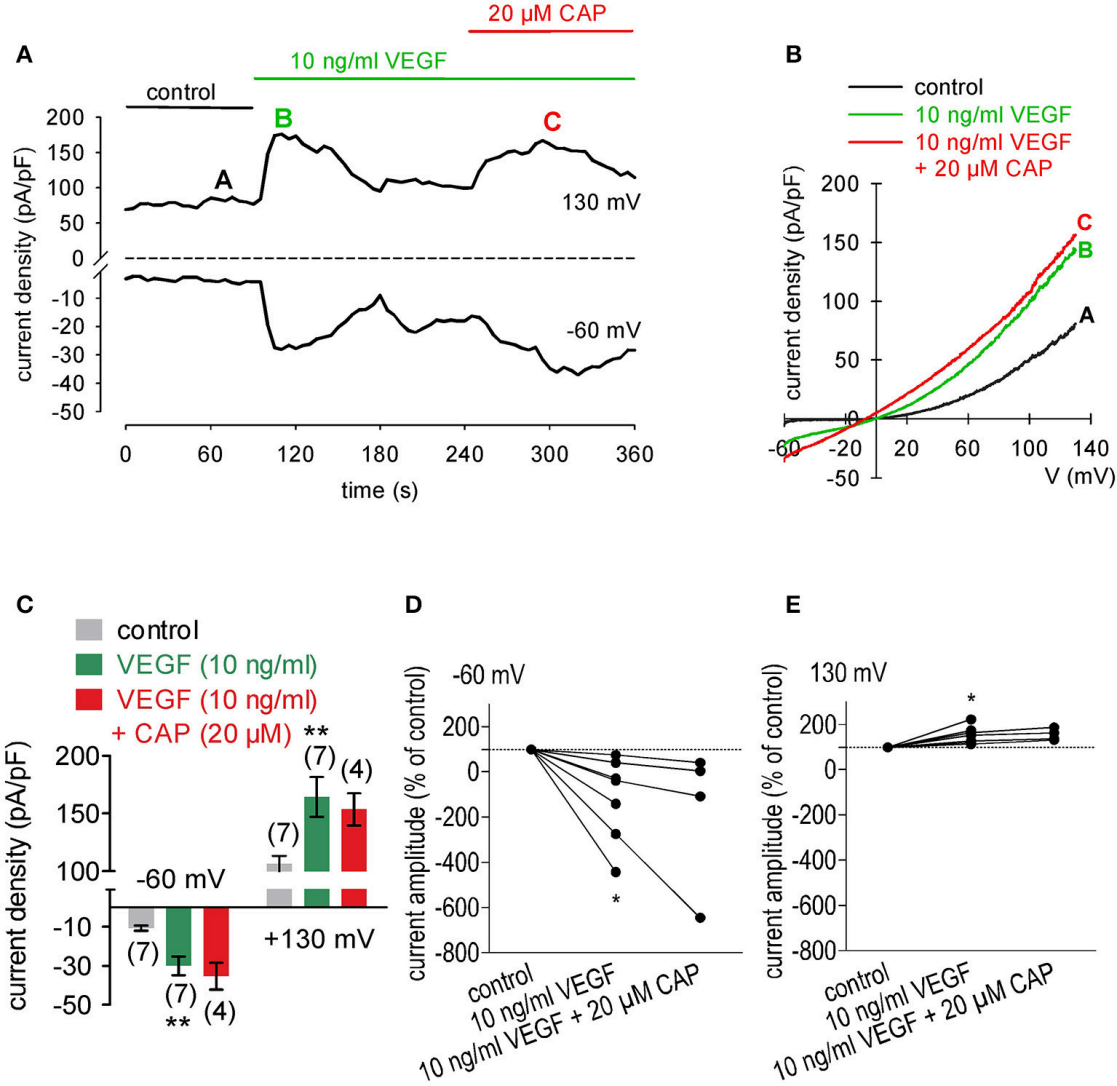
CAP does not augment increases in whole-cell currents in VEGF treated UM 92.1 cells. **(A)** Time course recording showing the current peak induced by 10 ng/ml VEGF and current peak after application of 20 μM CAP. **(B)** Original traces of VEGF- and CAP-induced current responses to voltage ramps. Current densities are shown before application (labeled as **A**), during application of VEGF (labeled as **B**), and after addition of CAP (labeled as **C**). **(C)** Summary of patch-clamp experiments with VEGF and CAP. The asterisks (**) indicate statistically significant increase with VEGF (*n* = 7; *p* < 0.01; paired tested) and unchanged magnitude of currents in the presence of CAP (*n* = 4; *p* > 0.05; paired tested). **(D)** Maximal negative current amplitudes induced by a voltage step from 0 to −60 mV are depicted in percent of control values before application of 10 ng/ml VEGF. VEGF-induced inward currents (*n* = 7; *p < 0.05) were not increased in the presence of 20 μM CAP (*n* = 4; *p* > 0.05). **(E)** Maximal positive current amplitudes induced by a voltage step from 0 to +130 mV are depicted in percent of control values before application of 10 ng/ml VEGF. VEGF-induced outwardly rectifying currents (*n* = 7; *p < 0.05) were not increased in the presence of 10 μM CAP (*n* = 4; *p* > 0.05).

As VEGF induces downstream signaling through transactivating TRPV1, we determined if CAP augmented VEGF induced TRPV1 activation. The results shown in Figures 4A,B indicate after application of VEGF, the maximal inward— and outward currents were −30 ± 5 pA/pF and 164 ± 17 pA/pF respectively (*n* = 7). Subsequently CAP failed to significantly enhance the whole-cell currents, which stabilized at − 35 ± 7 pA/pF and 154 ± 14 pA/pF respectively (*n* = 4; *p* > 0.05; Figures 4A,C–E).

### 3-T_1_AM activates TRPM8 in UM 92.1 cells

**Figure 5:**
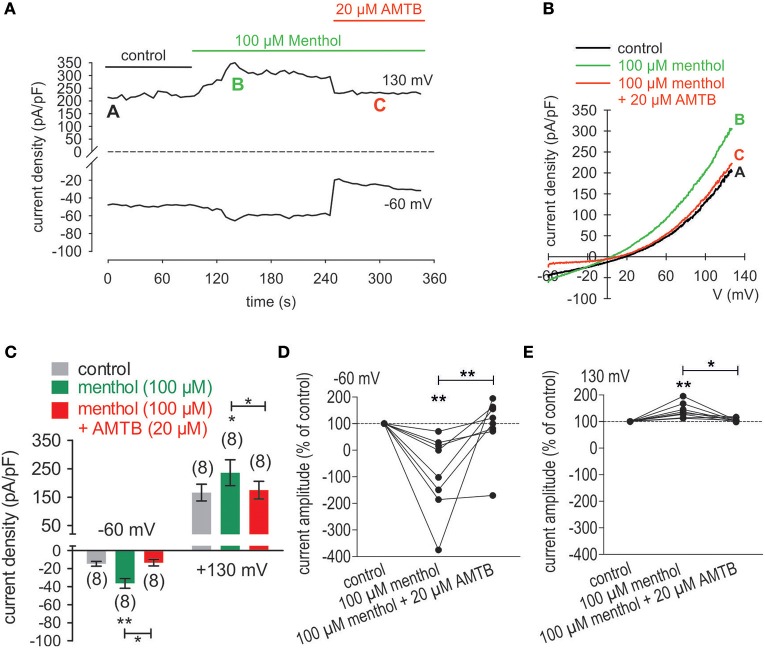
AMTB suppresses menthol-induced rises in whole-cell currents in UM 92.1 cells. **(A)** Time course recording showing the current increases induced by menthol (100 μM) and reduction after application of 20 μM AMTB. **(B)** Original traces of menthol-induced current responses to voltage ramps. Current densities are shown before application (labeled as **A**), during application of menthol (labeled as **B**), and after addition of AMTB (labeled as **C)**. **(C)** Summary of the patch-clamp experiments with menthol and AMTB. The asterisks (*) indicate statistically significant differences of in- and outward currents with menthol without AMTB (n = 8; inward currents; **p < 0.01; outward currents; *p < 0.05; paired tested) and in the presence of AMTB (*n* = 8; in- and outward currents *p < 0.05; paired tested). **(D)** Maximal negative current amplitudes induced by a voltage step from 0 to −60 mV are depicted in percent of control values before application of 100 μM menthol. Menthol-induced inward currents (*n* = 8; **p < 0.01) were clearly suppressed in the presence of 20 μM AMTB (*n* = 8; **p < 0.01). **(E)** Maximal positive current amplitudes induced by a voltage step from 0 to +130 mV are depicted in percent of control values before application of 100 μM menthol. Menthol-induced outwardly rectifying currents (*n* = 8; **p < 0.01) were clearly suppressed in the presence of 20 μM AMTB (*n* = 8; *p < 0.05).

As a positive control, the effect of 100 μM menthol, a highly selective TRPM8 agonist, was determined on whole-cell currents since this concentration was previously used to validate functional TRPM8 expression (Knowlton et al., 2011; Hirata and Oshinsky, 2012; Robbins et al., 2012; Mergler et al., 2013). As shown in Figures 5A,B, menthol increased the inward currents from −15 ± 3 pA/pF (control) to −36 ± 5 pA/pF (*p* < 0.01; *n* = 8; Figure 5C) whereas 20 μM AMTB suppressed this rise to −13 ± 4 pA/pF (*p* < 0.05; *n* = 8; Figure 5C). Similarly menthol increased the currents from 166 ± 30 pA/pF (control) to 236 ± 46 pA/pF, which AMTB suppressed to 175 ± 31 pA/pF (Figure 5C). The results of current normalization shown in Figures 5D,E (control set to 100%) affirm cell membrane delimited functional TRPM8 expression.

**Figure 6:**
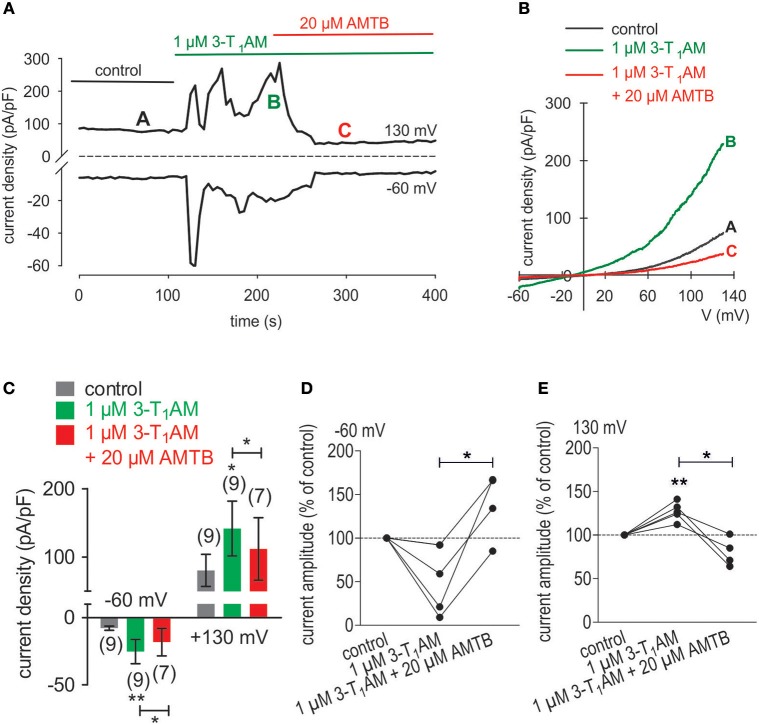
3-T_1_AM elicits increases in whole-cell currents through TRPM8 channels in UM 92.1 cells. **(A)** Time course recording showing the current increases induced by 3-T_1_AM and reduction after application of 20 μM AMTB. **(B)** Original traces of 3-T_1_AM -induced current responses to voltage ramps. Current densities are shown before application (labeled as **A**), during application of 3-T_1_AM (labeled as **B**), and after addition of AMTB (labeled as **C**). **(C)** Summary of patch-clamp experiments with 3-T_1_AM and AMTB. The asterisks (**) indicate statistically significant increase with 3-T_1_AM (*n* = 9; *p* < 0.05 at the minimum; paired tested) and decreases in the presence of AMTB (*n* = 7; *p* < 0.05; paired tested).). **(D)** Maximal negative current amplitudes induced by a voltage step from 0 to −60 mV are depicted in percent of control values before application of 1 μM 3-T_1_AM. 3-T_1_AM-induced inward currents were clearly suppressed in the presence of 20 μM AMTB (*n* = 4; *p < 0.05). **(E)** Maximal positive current amplitudes induced by a voltage step from 0 to +130 mV are depicted in percent of control values before application of 1 μM 3-T_1_AM. 3-T_1_AM-induced outwardly rectifying currents (*n* = 5; **p < 0.01) were clearly suppressed in the presence of 20 μM AMTB (*n* = 4; *p < 0.05).

Irrespective of 3-T_1_AM ranging from 200 nM to 10 μM, its effects were essentially the same as those obtained with menthol (Figures 6A,B). The largest increases were obtained over a range between 1 and 5 μM (Lucius et al., 2016). In UM 92.1 cells, 1 μM 3-T_1_AM increased the inward currents from −8 ± 2 pA/pF (control) to −25 ± 9 pA/pF (*p* < 0.01; *n* = 9; Figure 6C) whereas 20 μM AMTB suppressed this rise to −18 ± 10 pA/pF (*p* < 0.05; *n* = 7; Figure 6C). Similarly, 3-T_1_AM also increased the outward currents from 80 ± 24 pA/pF (control) to 142 ± 40 pA/pF, which AMTB suppressed to 112 ± 45 pA/pF (*n* = 7–9; *p* < 0.05) (Figure 6C). Similar results were obtained following current normalization shown in Figures 6D,E.

### 3-T_1_AM suppresses VEGF-induced rises in whole-cell currents

**Figure 7:**
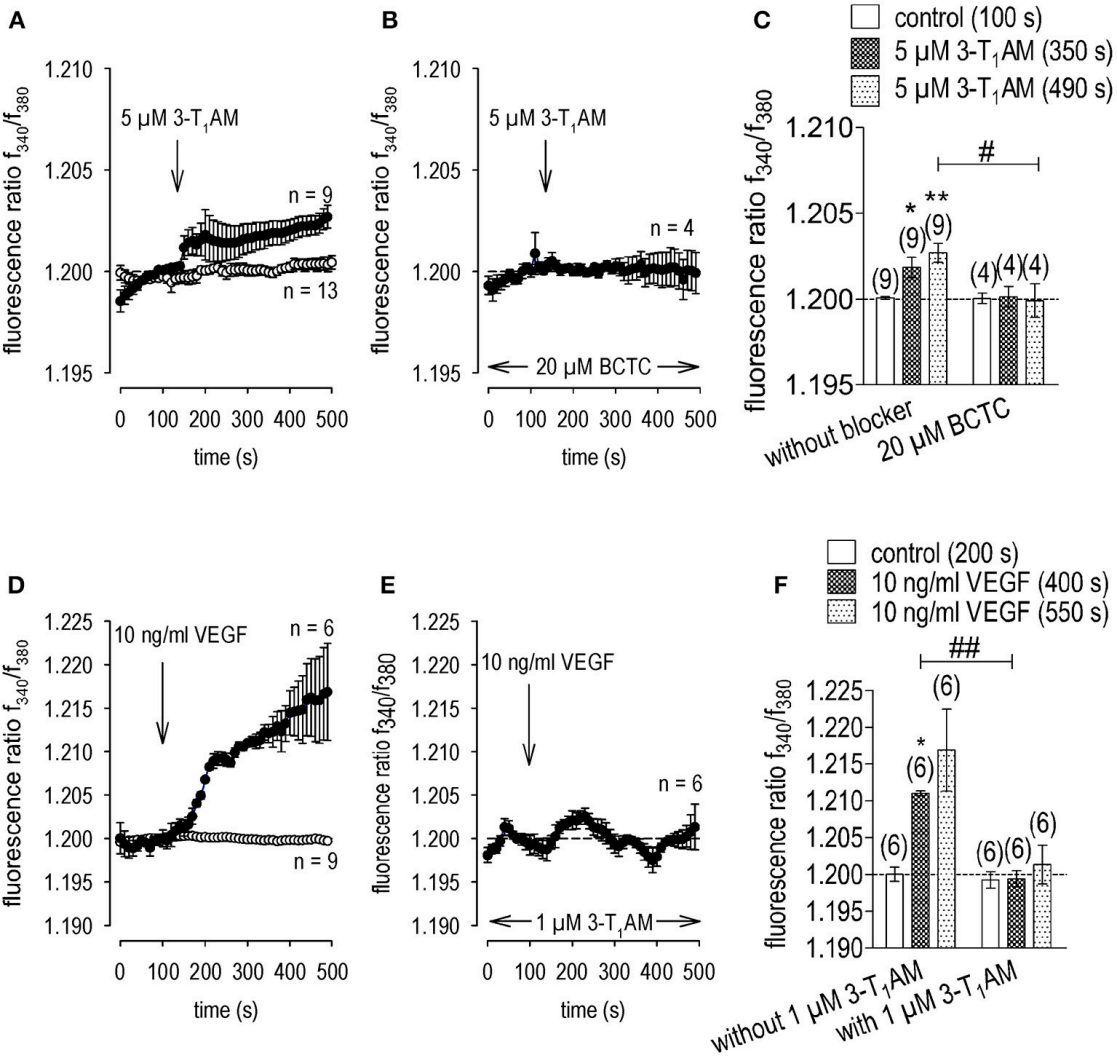
3-T_1_AM elicits increases in Ca^2+^ entry through TRPM8 channels but instead suppresses VEGF-induced Ca^2+^ increases in UM 92.1 cells. 3-T_1_AM (5 μM) or VEGF (10 ng/ml) was added at the time points indicated by arrows. Data are mean ± SEM of 4–9 experiments. **(A)** Mean trace showing 3-T_1_AM-induced Ca^2+^ increase (*n* = 9) whereas non-treated control cells showed a constant Ca^2+^ baseline (*n* = 13). **(B)** Same experiment as shown in **(A)**, but in the presence of BCTC (20 μM). BCTC clearly suppressed the 3-T_1_AM-induced Ca^2+^ increase (*n* = 4). **(C)** Summary of the experiments with 3-T_1_AM and BCTC. The asterisks (**) show significant Ca^2+^ increases with 3-T_1_AM (*n* = 9; 350 s; *p < 0.05; 490 s; **p < 0.01; paired tested). The hashtag (#) indicates a statistically significant difference of fluorescence ratios between 3-T_1_AM with and without BCTC (*n* = 6; 490s; ^#^*p* < 0.05; unpaired tested). **(D)** Mean trace showing VEGF-induced Ca^2+^ increase (*n* = 6) whereas non-treated control cells showed a constant Ca^2+^ baseline (*n* = 9). **(E)** Same experiment as shown in **(D)**, but in the presence of 3-T_1_AM (1 μM). 3-T_1_AM clearly suppressed the VEGF-induced Ca^2+^ increase (*n* = 6). **(F)** Summary of the experiments with VEGF and 3-T_1_AM. The asterisk (*) shows a significant Ca^2+^ increase with VEGF (*n* = 6; 400 s; *p < 0.05; paired tested). The hashtags (##) indicate statistically significant differences of fluorescence ratios between VEGF with and without 3-T_1_AM (*n* = 6; 400 s; ^*##*^*p* < 0.01; unpaired tested).

**Figure 8:**
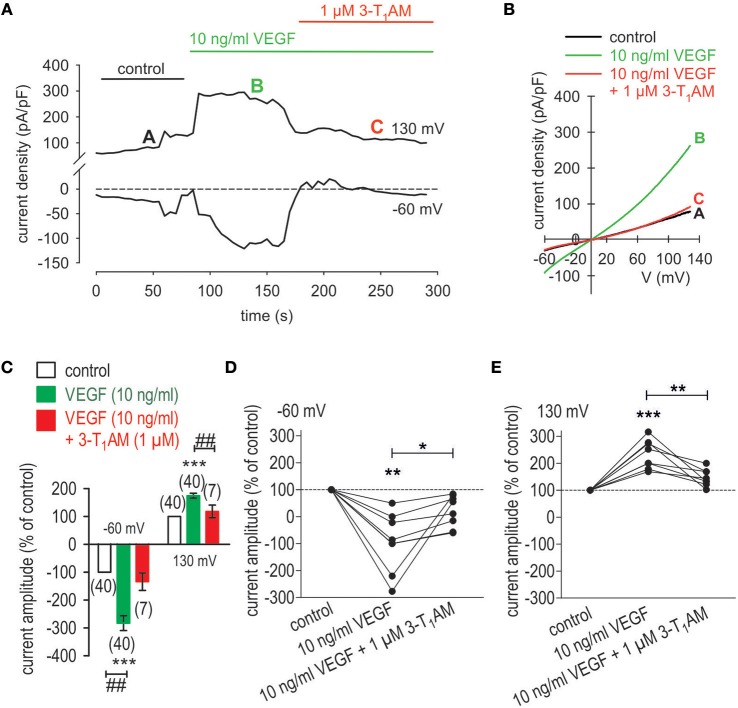
3-T_1_AM suppresses VEGF-induced rises in whole-cell currents in UM 92.1 cells. **(A)** Time course recording showing the current increases induced by VEGF (10 ng/ml) and reduction after application of 10 μM 3-T_1_AM. **(B)** Original traces of VEGF-induced current responses to voltage ramps. Current densities are shown before application (labeled as **A**), during application of VEGF (labeled as **B**), and after addition of 3-T_1_AM (labeled as **C**). Current densities as function of voltage were derived from the traces shown in panel **A**. **(C)** Summary of patch-clamp experiments with VEGF and 3-T_1_AM. The asterisks (***) indicate statistically significant increase with VEGF (*n* = 40; ***p < 0.005; paired tested). The hashtags (##) indicate statistically significant differences of whole-cell in- and outward currents between VEGF with and without 3-T_1_AM (*n* = 7–40; ^*##*^*p* < 0.01; unpaired tested). **(D)** Maximal negative current amplitudes induced by a voltage step from 0 to −60 mV are depicted in percent of control values before application of 10 ng/ml VEGF. VEGF-induced inward currents (*n* = 7; **p < 0.01) were clearly suppressed in the presence of 1 μM 3-T_1_AM (*n* = 7; *p < 0.05). **(E)** Maximal positive current amplitudes induced by a voltage step from 0 to +130 mV are depicted in percent of control values before application of 10 ng/ml VEGF. VEGF-induced outwardly rectifying currents (*n* = 7; ***p < 0.005) were clearly suppressed in the presence of 1 μM 3-T_1_AM (*n* = 7; **p < 0.01).

In TRPM8 transfected cells, 3-T_1_AM and BCTC increased and inhibited Ca^2+^transients, respectively (Lucius et al., 2016). These opposing effects were used to determine if TRPM8 activation suppresses CAP induced rises in TRPV1 activity whereas BCTC reduces the inhibitory effect of 3-T_1_AM on these responses to CAP. 3-T_1_AM (5 μM) induced a [Ca^2+^]_i_ transient (p < 0.01; *n* = 9; Figures 7A,C) whereas 20 μM BCTC inhibited this response (p < 0.05; *n* = 4; Figures 7B,C). Even though BCTC is reportedly as a mixed TRPM8/ TRPV1 antagonist in some cell types, it did not alter Ca^2+^ transients induced by a relatively high CAP concentration in a heterologous expression system (Lucius et al., 2016). 3-T_1_AM suppressed the 10 ng/ml VEGF-induced Ca^2+^ transient (Figures 7E,F). Another indication of suppression by TRPM8 of VEGF transactivation by TRPV1 is that 1 μM 3-T_1_AM suppressed VEGF-induced increases in the whole-cell currents (*n* = 7; *p* < 0.01; Figure 8), Mimicking of this inhibitory effect by icilin validates that 3-T_1_AM is a selective TRPM8 agonist (Khajavi et al., 2015, 2017; Lucius et al., 2016; Schanze et al., 2017).

### Icilin and menthol mimic suppression by 3-T_1_AM of VEGF transactivation of TRPV1

**Figure 9:**
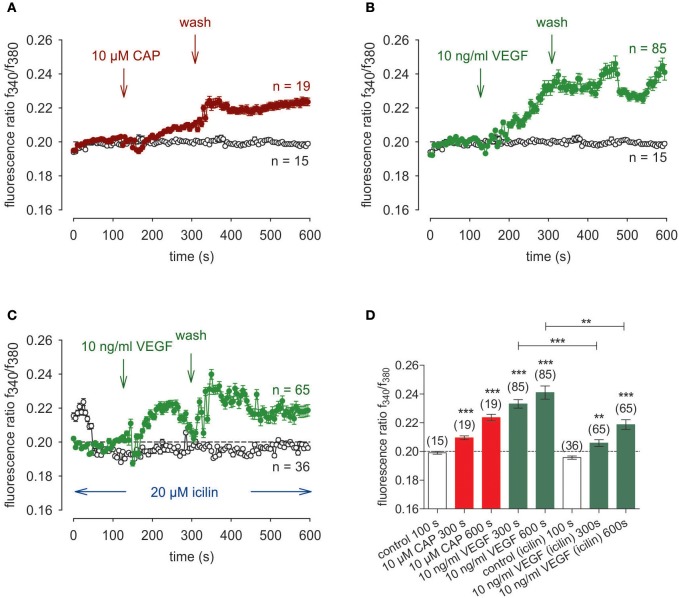
Icilin suppressed VEGF-induced Ca^2+^ increase in UM 92.1 cells. Drugs were added at the indicated time points (arrows). Data are mean ± SEM of 15–85 Ca^2+^ traces in each set of experiments. **(A)** CAP (10 μM) induced an irreversible Ca^2+^ increase (*n* = 19). A washout did not reduce the Ca^2+^ levels. Non-treated control cells showed a constant Ca^2+^ baseline (*n* = 15). **(B)** The similar Ca^2+^ response pattern could be observed with 10 ng/ml VEGF instead of CAP (*n* = 85). **(C)** Same experiment as shown in **(B)**, but in the presence of icilin (10 μM) (*n* = 65). Icilin partially suppressed the VEGF-induced Ca^2+^ increase (*n* = 65). Non-treated control cells showed a constant Ca^2+^ baseline in the presence of icilin (*n* = 36). **(D)** Summary of the experiments with CAP, VEGF and icilin. The asterisks (***) show significant Ca^2+^ increases with CAP and VEGF (n = 15–85; **p < 0.01 at the minimum; unpaired tested).

**Figure 10:**
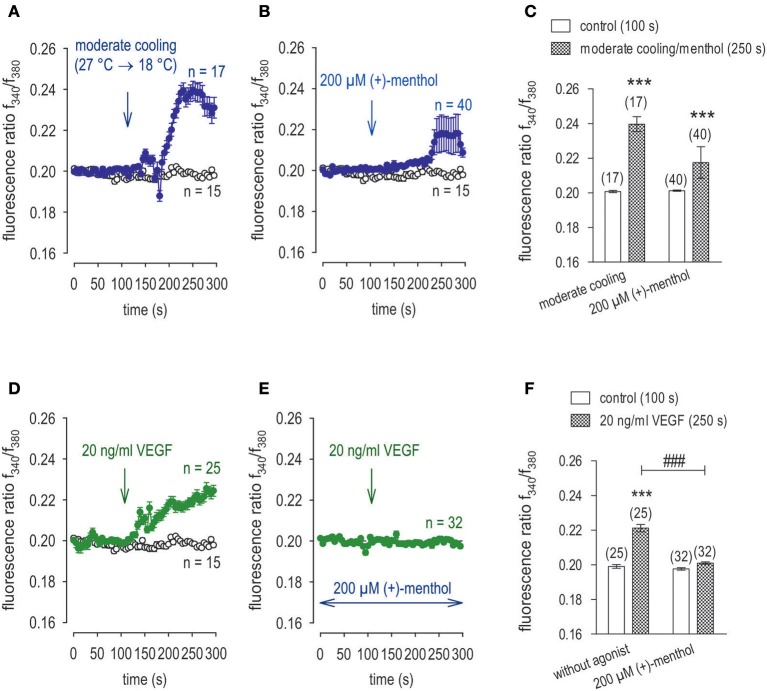
Menthol suppressed VEGF-induced Ca^2+^ increase in UM 92.1 cells. Drugs were added at the indicated time points (arrows). Data are mean ± SEM of 15–40 Ca^2+^ traces in each set of experiments. **(A)** Moderate cooling (≈27 to 18°C) induced a Ca^2+^ increase, which partially recovered (*n* = 17). Non-treated control cells showed a constant Ca^2+^ baseline (*n* = 15). **(B)** TRPM8 activation by menthol (200 μM) led to a Ca^2+^ increase, which is at lower levels compared to moderate cooling (*n* = 40). **(C)** Summary of the experiments with cooling and menthol. The asterisks (*) show significant Ca^2+^ increases with moderate cooling and menthol (*n* = 17 - 40; ***p < 0.005; paired tested). **(D)** The Mean trace showing VEGF-induced Ca^2+^ increase (*n* = 25). **(E)** Same experiment as shown in **(D)**, but in the presence of menthol (200 μM). Menthol clearly suppressed the VEGF-induced Ca^2+^ increase (*n* = 32). **(F)** Summary of the experiments with VEGF and menthol. The asterisks (*) show significant Ca^2+^ increases with VEGF (*n* = 25; ***p < 0.005; paired tested). The hashtags (###) show significant Ca^2+^ decreases in the presence of menthol (*n* = 32; ^###^*p* < 0.005; unpaired tested).

As a positive control, the effects of CAP and VEGF were determined using an alternative fluorescence Ca^2+^ imaging data acquisition method as described in the method section. As shown in Figure 9A, a reduced CAP concentration (10 μM) led to an increase of f_340nm_/f_380nm_ from 0.2021 ± 0.0004 (100 s) to 0.2094 ± 0.0013 (300 s) (*n* = 19; *p* < 0.005) whereas a washout did not reduce the Ca^2+^ level. With 10 ng/mg VEGF instead of CAP (Figure 9B), this ratio increased from to 0.2011 ± 0.0004 (100 s) to 0.2331 ± 0.0029 (300 s) (*n* = 85; *p* < 0.005) and a washout did not augment this response Ca^2+^ transient (Figures 9B,C). However, preincubation of the cells with icilin suppressed the VEGF-induced increase to 0.2058 ± 0.0023 at 300 s (*p* < 0.005) and to 0.2187 ± 0.0034 at 600 s (both *n* = 65; *p* < 0.01) (Figures 9C,D). Menthol had the same inhibitory effect as icilin. Functional TRPM8 expression was validated based on a correspondence between the transients induced by cooling from 20 to 18°C (Figures 10A–C) and exposure to 200 μM menthol (Figures 10B,C). Furthermore, as with icilin, preincubation of the cells with menthol suppressed the VEGF-induced Ca^2+^ transient even at a higher VEGF concentration since 20 ng/ml VEGF increased the f_340nm_/f_380nm_ ratio from 0.1991 ± 0.011 (100 s) to 0.2212 ± 0.0021 (250 s) (*n* = 25; *p* < 0.005) (Figure 10D). In contrast, 200 μM menthol completely blocked this effect (e.g., f_340nm_/f_380nm_ = 0.2009 ± 0.0007 at 250 s; *n* = 32; *p* < 0.005) (Figures 10E,F). In summary, the near equivalence between the transients induced by either icilin, menthol, or 3-T_1_AM and their inhibitory effects on VEGF-induced TRPV1 transactivation confirms that this thyroid hormone metabolite is a selective TRPM8 agonist.

### Cannabinoid receptor type 1 activity modulates inhibition of TRPV1 by 3-T_1_AM

**Figure 11:**
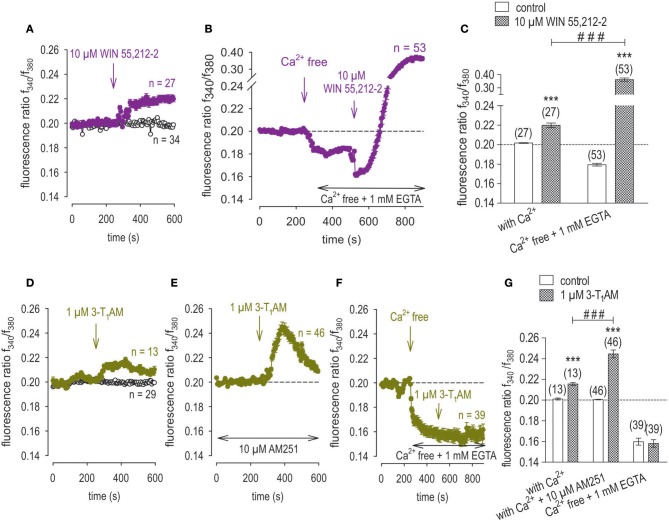
3-T_1_AM modulation of TRPV1 is associated with cannabinoid receptor type 1 activity. Drugs were added at the indicated time points (arrows). Data are mean ± SEM of 13–53 Ca^2+^ traces in each set of experiments. **(A)** WIN 55,212-2 (10 μM) induced a Ca^2+^ increase, (*n* = 27). Non-treated control cells showed a constant Ca^2+^ baseline (*n* = 34). **(B)** Ca^2+^ free condition reduced the intracellular Ca^2+^ level (baseline) and extracellular application of WIN 55,212-2 (10 μM) strongly increased the intracellular Ca^2+^ level (*n* = 53). **(C)** Summary of the experiments with WIN 55,212-2 with and without external Ca^2+^. The asterisks (***) show significant Ca^2+^ increases with WIN 55,212-2 (*n* = 27 - 53; ***p < 0.005; paired tested). The hashtags (###) show significant stronger Ca^2+^ increase under external Ca^2+^ free conditions (*n* = 53; ^###^*p* < 0.005; unpaired tested). **(D)** Mean trace showing 3-T_1_AM-induced Ca^2+^ increase (*n* = 13) whereas non-treated control cells showed a constant Ca^2+^ baseline (*n* = 29). **(E)** Same experiment as shown in (D), but in the presence of AM251 (10 μM) (*n* = 46). The 3-T_1_AM-induced Ca^2+^ increase is at higher levels compared to the effect without the CB1 blocker. **(F)** Same experiment as shown in **(B)**, but with 1 μM 3-T_1_AM instead of WIN 55,212-2. 3-T_1_AM did not change the intracellular Ca^2+^ concentration. **(G)** Summary of the experiments with 3-T_1_AM with and without AM251 or external Ca^2+^. The asterisks (***) show significant Ca^2+^ increases with 3-T_1_AM (*n* = 13–46; ***p < 0.005; paired tested). The hashtags (###) show significant difference of the 3-T_1_AM-induced Ca^2+^increase with and without AM251 (*n* = 13–46; ^###^*p* < 0.005; unpaired tested).

Since the G protein-coupled cannabinoid receptor 1 (CB1) is expressed in uveal melanoma cells (Mergler et al., 2014), we determined if either this receptor or its coupled G-proteins affect interactions between TRPM8 and TRPV1 in UM cells. To deal with this question, the individual effects of the selective CB1 receptor antagonist, AM251, and a corresponding agonist, WIN 55,212-2 (both 10 μM) were characterized by measuring their effects on [Ca^2+^]_i_ in UM 92.1 cells. WIN 55,212-2 induced a Ca^2+^ transient at a different cell passage compared to our previous studies (*n* = 27; *p* < 0.005). This validates CB1 involvement in Ca^2+^ regulation in UM 92.1 cells (Figure 11A). Interestingly, the WIN-induced Ca^2+^ increase was at significant higher levels under Ca^2+^ free conditions (*n* = 53; *p* < 0.005) (Figures 11B,C). Similarly, 1 μM 3-T_1_AM also induced such a response. On the other hand, preincubation of the cells with the CB1 blocker AM251 (10 μM) augmented this rise induced by 3-T_1_AM. The transient reached with 3-T_1_AM by itself was 0.2155 ± 0.0014 (*n* = 13) at 400 s only whereas with AM251 (10 μM) it rose to 0.2446 ± 0.0037 at the same time (*n* = 46; *p* < 0.005) (Figures 11D,E,G). In contrast, 3-T_1_AM failed to induce a transient under Ca^2+^ free conditions (*n* = 39) (Figures 11F–G). In summary, changes in CB1 activity and/or coupled G-protein activity modulate interactions between TRPV1 and TRPM8.

## Discussion

We describe here TRPV1 and TRPM8 functional activity and their interactions in modulating VEGF-induced signaling in UM cells. Even though a short-term UM cell culture (*P* < 20) was used in this study, variations may occur in gene expression profiles between UM primary tumors and their derived cell lines. Nevertheless, there were only moderate modifications in the gene products (Mouriaux et al., 2016). Accordingly, Mouriaux et al. suggested that cell lines might represent useful tools in functional assays, as well as pharmacologic and genetic studies (Mouriaux et al., 2016). The TRPV1 and TRPM8 functional activity identified in these UM cells is consistent with the correspondence between the mRNA and protein expression patterns previously described in several other UM cell-line types (Mergler et al., 2014). Similarly, functional interaction between TRPM8 and TRPV1 is evident because TRPM8 activation inhibited increases in TRPV1 functional activity induced by CAP in both corneal epithelial and endothelial cells (Khajavi et al., 2015; Lucius et al., 2016). Furthermore, TRPM8 activation blunts transactivation of TRPV1 by VEGF in UM cells (Figures 7, 8). This modulation of VEGF-induced increases in Ca^2+^ influx mediated by TRPV1 activation shows that this receptor triad contributes through crosstalk to the growth promoting effects of VEGF in UM cells derived from malignant tumors. Such crosstalk is consistent with other studies wherein TRPM8 activation dampens CAP-induced TRPV1 activation by VEGF (Millqvist, 2016; Takaishi et al., 2016). This consistency in interactions among this receptor triad in different cell types suggests that results obtained with one cell type may be applicable to various cell types.

### TRPV1 functional expression evidence

Even though CPZ has limited selectivity as a TRPV1 antagonist (Docherty et al., 1997; Liu and Simon, 1997; Oh et al., 2001; Ray et al., 2003; Teng et al., 2004) and limited TRPM8 selectivity (Behrendt et al., 2004; Xing et al., 2007; Mergler et al., 2013), its suppression of CAP-induced [Ca^2+^]_i_ transients and whole-cell currents in UM 92.1 cells are indicative of TRPV1 functional activity (Figures 2, 3). Its functional expression was also clearly detectable in both primary cultivated PM and healthy human uveas (Mergler et al., 2014). However, TRPV1 receptor density was probably at lower levels because of more extensive data scatter and a delayed response to CAP in most PM measurements (Figure 1B). Nevertheless, the maximal rises in Ca^2+^ influx in normal PM cells closely correspond to those in UM 92.1 cells.

The dynamic range of our Ca^2+^ imaging system was limited to 0.20 for detecting increases in the fluorescence ratio because the initial fluorescence responses of the two exciting wavelengths were at a relatively high level, which compressed the dynamic range of our measurements due to mathematical reasons. Therefore, even ratio changes of only 0.015 for CAP were significant and the measurements were clearly discernible and reproducible (Figure 1A). Another indication of the adequate resolving power of our measurements is that the effects of CAP and icilin especially in UM 92.1 cells were irreversible and reached a steady state in most experiments. Similarly, this irreversibility was described in other eye tumor cells (Mergler et al., 2012a, 2014; Garreis et al., 2016) as well as healthy eye surface cells (Khajavi et al., 2015; Lucius et al., 2016).

### Different TRPM8 expression levels in UM 92.1 cells and PM

In healthy human uveas, TRPM8 mRNA expression was not evident whereas the TRPA1 PCR signal was present at very high levels (Mergler et al., 2014). On the other hand, icilin (Rawls et al., 2007) increased Ca^2+^ transients in UM 92.1 cells (Figure 1D) whereas this effect was not evident in PM (Figures 1E,F). Even though menthol activates TRPM8 (Eccles, 1994; Galeotti et al., 2002; Bautista et al., 2007; Pedretti et al., 2009), the magnitudes of these transients did not correlate with the TRPM8 expression levels in certain cancer cells indicating a TRPM8-independent signaling pathway (Naziroglu et al., 2018). Irrespective of that, menthol also activated TRPM8 in the absence of extracellular Ca^2+^, whereas responses to icilin are Ca^2+^ dependent (McKemy et al., 2002; Chuang et al., 2004). Therefore, icilin appears to induce Ca^2+^ transients by increasing Ca^2+^ influx from the extracellular medium (McKemy et al., 2002; Andersson et al., 2004; Behrendt et al., 2004). Nevertheless, TRPA1 involvement cannot be excluded because icilin can also interact with TRPA1 even though icilin was relatively ineffective at inducing Ca^2+^ transients and there is no detectable TRPA1 mRNA expression in this cell type (Mergler et al., 2014). Therefore, TRPM8 activity in PM and human uveas is relatively low compared to higher levels in malignant uveal cell types (Mergler et al., 2014). In contrast, the CAP-induced Ca^2+^ transients were comparable suggesting no difference in TRPV1 expression levels in these two different cell types (Figure 1B).

### Crosstalk between VEGFR and TRPV1

VEGF transactivated TRPV1 through its interaction with VEGFR since the Ca^2+^ transients and their underlying currents were both fully blocked during exposure to CPZ. Furthermore, the stimulation by VEGF of TRPV1 was maximal since supplementation with CAP failed to augment the increases in whole-cell currents induced by VEGF application (Figures 4, 7, 8).

Unlike with CPZ, the TRPM8 blocker AMTB (Lashinger et al., 2008) failed to suppress these transients induced by VEGF suggesting that VEGFR solely transactivates TRPV1 (Figure 2). This agrees with what was described in a benign tumor (Garreis et al., 2016). The smaller inhibitory effects of BCTC on VEGF-induced increases in Ca^2+^ influx than those induced by CPZ are supportive of the notion that BCTC is a mixed TRPV1/TRPM8 antagonist (Behrendt et al., 2004; Weil et al., 2005; Vriens et al., 2009; Benko et al., 2012; Liu et al., 2016). In contrast, AMTB is a more selective TRPM8 antagonist (Lashinger et al., 2008) since it failed to block VEGF-induced transactivation of TRPV1 (Figures 2D, 3A,B). The limited specificity of BCTC as a TRPV1 blocker is supported by our finding that at a relatively high CAP concentration (20 μM), BCTC was ineffective as a blocker of TRPV1 activation in human corneal epithelial cells (Lucius et al., 2016). The marked difference between the large inhibitory effect of CPZ and the minimal effect of AMTB on increases in currents induced by VEGF confirms that VEGF solely transactivates TRPV1 (Figure 3). However, VEGFR is also known to regulate multiple channels including TRPs (Garnier-Raveaud et al., 2001; Hamdollah Zadeh et al., 2008; Thilo et al., 2012; Reichhart et al., 2015; Wu et al., 2015; Qin et al., 2016). Specifically, McNaughton et al. demonstrated that nerve growth factor (NGF) receptors in HEK293 cells transfected with plasmids containing cDNAs coding for TRPV1 and for the Tropomyosin receptor kinase A (TrkA) receptor for NGF increased the expression level of TRPV1 but did not sensitize or activate the receptor (Zhang et al., 2005; Vay et al., 2012). One explanation may be that NGF is different from VEGF or that our study used non-excitable (tumor) cells.

### TRPM8 activation suppresses VEGF-induced rises in Ca^2+^ influx

3-T_1_AM suppressed VEGFR transactivation of TRPV1 (Figures 7D–F, 8) was blocked by BCTC in TRPM8-transfected cells, in corneal and conjunctival epithelial cells derived from normal cells (Khajavi et al., 2015; Lucius et al., 2016) and in UM 92.1 cells (Figures 7, 8) as well as in thyroid PCCL3 cells (Schanze et al., 2017). These effects were similar to those induced by AMTB, which is consistent with significant antagonism by BCTC of TRPM8 (Figures 5, 6, 7A–C).

**Figure 12:**
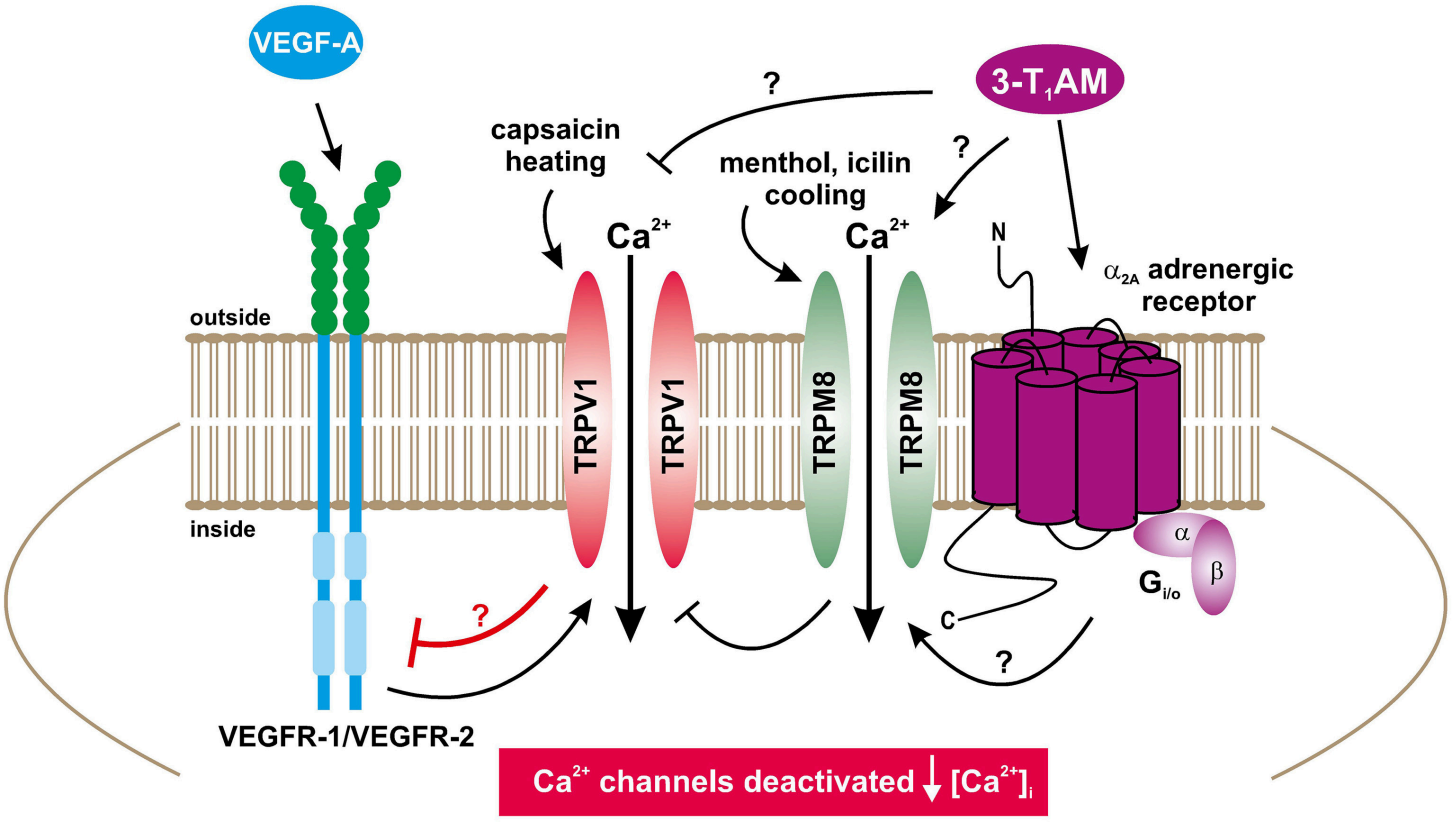
Suggested Ca^2+^ signal transduction model accounting for how TRPM8 activation affects receptor-linked signaling pathways (Mergler et al., 2014; Khajavi et al., 2017). Ca^2+^ channels such as TRPs of the TRPV1 subtype (capsaicin receptor) can be selectively activated by heat (>43°C) or capsaicin and blocked by CPZ (Figure 1A) (Mergler et al., 2014). VEGF-A activating VEGFR-1/VEGFR-2 can also activate TRPV1 (Figures 2, 3). The TRPM8 subtype (menthol receptor) can be selectively activated by cold (23–28°C), menthol, icilin or 3-T_1_AM and blocked by BCTC/AMTB (Figures 1D, Figures 5, 6) (Mergler et al., 2014; Khajavi et al., 2017). Notably, a G-protein coupled receptor (GPCR) coupled to Gi/o proteins could be activated by 3-T_1_AM (Dinter et al., 2015a; Khajavi et al., 2017; Schanze et al., 2017). 3-T_1_AM may also directly activate TRPM8 by a GPCR-independent mechanism (↑[Ca^2+^]_i_]) (Khajavi et al., 2017). If TRPM8 is activated by 3-T_1_AM, 3-T_1_AM suppresses VEGRF via TRPV1 (Figures 7, 8). Notably, 3-T_1_AM may also directly suppress TRPV1 via VEGFR by a GPCR-independent mechanism (↓[Ca^2+^]_i_]) (Figure 8) Menthol and icilin mimic the 3-T_1_AM effect and suppresses increases in TRPV1 activity by VEGF (Figures 9, 10).

The 3-T_1_AM mediated Ca^2+^ transients as well as increases in their underlying currents occurred at lower concentrations in UM 92.1 melanoma cells than those in healthy cells or thyroid cells (Khajavi et al., 2017; Schanze et al., 2017). Specifically, 3-T_1_AM was used over a concentration range from 0.2 to 10 μM with 1–5 μM having maximal stimulatory effects on whole-cell currents, which agrees with previous studies using corneal epithelial cells (Lucius et al., 2016). 3-T_1_AM had a concentration dependent inhibitory effect on Ca^2+^ transients that may be attributable to different modes of action. With 0.5 μM 3-T_1_AM, only whole-cell currents increased without any inhibitory effect on VEGF-induced rises in Ca^2+^ influx (data not shown). However, with 1 μM 3-T_1_AM VEGF-induced rises in Ca^2+^ influx also declined with a time course that was similar to that obtained with CPZ (Figures 2B, 7E, 11D). It is conceivable that the effect of 0.5 μM 3-T_1_AM is membrane delimited rather than causing release of Ca^2+^ from intracellular stores. Perhaps, 1 μM 3-T_1_AM is at a high enough concentration for it to gain access to cytosolic intracellular TRPV1 binding sites with which CPZ also binds? If this supposition is proven to be correct, 3-T_1_AM may have dual effects that include activating TRPM8 and at higher concentrations also suppressing TRPV1 activation induced by VEGF. As the effects of modulators of TRP channel activity on Ca^2+^ influx mirrored those on whole-cell currents (Figures 7, 8), this agreement supports the notion that 3-T_1_AM has a relevant role in regulating VEGF-mediated Ca^2+^ regulation in tumor cells. In addition, we could demonstrate that TRPM8 activation by menthol or icilin mimic the 3-T_1_AM effect and is able to suppress increases in TRPV1 activity by VEGF (Figures 9, 10). To the best of our knowledge, this report is the first one describing such control in both benign eye surface tumor cells (Garreis et al., 2016) and now in metastatic UM 92.1 cells. On the other hand, the possible Ca^2+^ signal transduction pathways activated by 3-T_1_AM as a specific activator of TRPM8 may be more complex as suggested in Figure 12 (Khajavi et al., 2017).

There are numerous studies showing that 3-T_1_AM also regulates beta-adrenergic receptors, trace amine-associated receptor 2, muscarinic receptors, and K^+^ channels (Scanlan et al., 2004; Ghelardoni et al., 2009; Panas et al., 2010; Cichero et al., 2014; Dinter et al., 2015b; Hoefig et al., 2016). 3-T_1_AM has also been described as an antagonist of muscarinic type 3 receptor (Laurino et al., 2016). Furthermore, beta-adrenergic receptors and muscarinic receptors are expressed in multiple melanoma cells including primary uveal melanoma (92.1, Mel202) (Janik et al., 2017). In addition, changes in K^+^ channel activity have been implicated in modulating progression of melanoma (Oppitz et al., 2008; Luo et al., 2017). These studies indicate that 3-T_1_AM may not be directly or solely targeting TRPM8 (Figure 12).

### G protein-coupled cannabinoid receptor-1 modulates 3-T_1_AM suppression of TRPV1 channels

It has been suggested that 3-T_1_AM is a multitarget ligand (Zucchi et al., 2014) interacting with different TRP channel subtypes including TRPM8 (Khajavi et al., 2015, 2017; Lucius et al., 2016). Since functional CB1 expression has been described in ocular tumor cells (Mergler et al., 2012a, 2014) and in healthy ocular cells (Stumpff et al., 2005; Yang et al., 2010, 2013), we determined if 3-T_1_AM interacts also with the cannabinoid receptor 1 CB1. CB1 activation by WIN 55,212-2 induced Ca^2+^transients, which were larger in a Ca^2+^ free conditions than with 2 mM external Ca^2+^ (Figures 11A–C). On the other hand, 3-T_1_AM failed to elicit a Ca^2+^ transient in a Ca^2+^ free medium (Figure 11F) whereas in the presence of AM251 and external Ca^2+^ in the medium 3-T_1_AM-induced Ca^2+^ transients that were larger in the presence of the CB1 blocker than in its absence (Figures 11D,E). Since CB1 activation also suppresses TRPV1 activation (Yang et al., 2013; Mergler et al., 2014), there may be an inverse relationship between increases in TRPM8 activity and declines in CB1 activity. Overall, the mechanisms involved in 3-T_1_AM modulation of TRPV1 channels may also involves contributions by other receptors such as CB1 and/or its coupled G-protein mediators.

### Clinical relevance

3-T_1_AM is a potential therapeutic agent for suppressing UM expansion as already indicated in other studies demonstrating that this thyroid hormone metabolite reduces cancer cell growth and viability (Rogowski et al., 2017; Shinderman-Maman et al., 2017). In UM cells, modulation of their metastatic activity appears to include changes in TRPV1 activity induced by TRPM8 and possibly CB1. The model for such control shown in Figure 12 proposes that VEGF secreted by UM cells stimulates intracellular Ca^2+^ influx in endothelial cells, which is a requisite for driving angiogenesis and promotes UM proliferation and metastasis. Since TRPM8 activation has an opposing effect on TRPV1 activity, targeting TRPM8 may provide an effective alternative to suppress metastatic melanoma progression without side effects. Such an approach appears to be tenable based on the fact that functional TRPM8 expression is only detectable in the UM cells rather than PM cells. There is an urgent need for further assessment of the validity of this option since there are no measures currently available to reverse melanoma metastasis.

## Author contributions

LW, CB, SM, and PR designed the study, analyzed the data, wrote and edited the manuscript. LW and CB contributed equally to the work. JK contributed with his expertise on thyroid hormone metabolites, discussed data and their interpretation and helped edit the manuscript. LW, CB, AB, PD, NL, MS, HvdW, IR, and SM performed calcium measurements and planar patch-clamp recordings as well as plot analyses. LW, CB, AB, PD, MS, HvdW, and SM created diagrams. SM created the schematic drawing.

### Conflict of interest statement

The authors declare that the research was conducted in the absence of any commercial or financial relationships that could be construed as a potential conflict of interest.

## Abbreviations

3-T_1_AM: 3-Iodothyronamine (endogenous thyroid hormone (TH)-derived metabolite) (Scanlan et al. 2004)
92.1: human uveal melanoma cell line 92.1 (De Waard-Siebinga et al. 1995)
AMTB: N-(3-Aminopropyl)-2-[(3-methylphenyl)methoxy]-N-(2-thienylmethyl)benzamide hydrochloride [TRPM8 blocker (Lashinger et al. 2008)]
BCTC: N-(4- tertiarybutyl-phenyl)-4-(3-chloropyridin-2-yl) tetrahydropyrazine-1(2H)-carboxamide
CAP: Capsaicin [TRPV1 agonist (Vriens et al. 2009)]
CB1: Cannabinoid receptor 1
CZP: Capsazepine [TRPV1 antagonist (Vriens et al. 2009)]
EGFR: Epidermal growth factor receptor
FDA: Food and Drug Administration
hTAAR1: Human trace amine-associated receptor
PM: Porcine melanocytes
RPE: Retinal pigment epithelium
TRPA: Transient receptor potential ankyrin
TRPC: Transient receptor potential canonical
TRPM: Transient receptor potential melastatin
TRPs: Transient receptor potential channels
TRPV: Transient receptor potential vanilloid
UM: Uveal melanoma
VEGF: Vascular endothelial growth factor
VEGFR: Vascular endothelial growth factor receptor
VPF: Vascular permeability factor.
